# Intuitive eating in the COVID-19 era: a study with university students in Brazil

**DOI:** 10.1186/s41155-024-00306-1

**Published:** 2024-07-25

**Authors:** Ana Corrêa Ruiz, Wagner de Lara Machado, Helen Freitas D’avila, Ana Maria Pandolfo Feoli

**Affiliations:** 1 Postgraduate Program in Health Promotion, University of Santa Cruz do Sul, Av. Independência, 2293, Santa Cruz do Sul, Brazil-RS 96815-900 Brazil; 2 Postgraduate Program in Psychology, Pontifícia Universidade Católica Do Rio Grande Do Sul, Av. Ipiranga, 6681-Partenon, Porto Alegre, Brazil-RS 90619-900 Brazil

**Keywords:** Students, Eating behavior, Eating and food intake disorders, Body mass index, COVID-19

## Abstract

**Background:**

The recurrence of weight gain is attributed to the homeostatic regulation of hunger and satiety signals, influenced by metabolic state, nutrient availability, and non-homeostatic mechanisms shaped by reinforced consequences from experiences. In response, Evelyn Tribole and Elyse Resch proposed Intuitive Eating (IE) in 1980, countering restrictive diets. IE, inversely correlated with Body Mass Index (BMI), binge eating, and anxiety/depression symptoms, fosters mind–body-food harmony by recognizing hunger and satiety cues. IE encourages meeting physiological, not emotional, needs, permitting unconditional eating, and relying on internal signals for food decisions. Amidst university students’ stress, exacerbated during the COVID-19 pandemic, understanding their eating behavior, particularly intuitive eating levels, becomes crucial.

**Objective:**

This study aimed to assess the IE level of Brazilian students during the COVID-19 pandemic.

**Methods:**

This cross-sectional study, the first to analyze the Intuitive Eating of students in Brazil during the pandemic, was conducted using an online questionnaire.

**Results:**

The sample comprised 1335 students, most of whom were women (82.17%), with a mean age of 26.12 ± 7.9 years, and a healthy nutritional status (57.58%). The mean IE score was 3.2 ± 0.6. A significant association was found between the confinement situation, the type of housing unit, and the IE subscale-Unconditional Permission to Eat (*p* = 0.043). However, there was no association between the other subscales and the total IE scale. Regarding self-reported mental and eating disorders, the most frequent were anxiety (21.2%), depression (6.5%), and binge eating disorder (BED) (4.7%). IE was negatively associated with BED (*B* =  − 0.66; *p* < .001), bulimia nervosa (*B* =  − 0.58; *p* < .001), body mass index (BMI) (*p* < .001) and self-reported anxiety (*B* =  − .102; *p* = 0.16). The male sex showed a higher IE score compared with the female sex (*p* < .001).

**Conclusion:**

While no significant association was found between IE and the confinement situation, a significant association was found between housing type and the Unconditional Permission to Eat subscale.

## Introduction

Individuals are born as intuitive eaters (Tribole & Resch, 2012); however, due to various factors over the course of life, they may lose this ability to connect with hunger and satiety cues, obesity is one such instance. This occurs because hunger and satiety signals are regulated by homeostatic mechanisms, which involve our metabolic state and the availability of nutrients, as well as by non-homeostatic mechanisms, involving a system that works through reinforcing consequences arising from our experiences (Alonso-Alonso et al., 2015). Therefore, when it comes to eating behavior, the decision of what to eat, when to eat, and how much to eat is not only related to physiological signals, but also involves social, cultural, environmental, and psychological factors (Alvarenga et al., 2018).

In 1980, Evelyn Tribole and Elyse Resch proposed a new eating style: intuitive eating (IE). This practice aims to establish harmony between the body, the mind, and food in order to lead to the recognition of hunger and satiety cues (Tribole & Resch, 2012) IE was inversely correlated with body mass index (BMI) and compulsive eating, restrictive eating, anxiety, and depression symptoms. In addition, people with greater interoceptive awareness and acceptance of body image had higher IE (Anderson et al., 2016; Camilleri et al., 2016; Linardon et al., 2021; Richard et al., 2019). Its 3 pillars are (a) eating to meet physiological rather than emotional needs; (b) unconditional permission to eat, and (c) relying on internal hunger and satiety cues to determine what, how much, and when to eat.

University students, who find themselves in the phase of young adulthood, endure a period marked by considerable stress (Acharya et al., 2018). This is attributed to a myriad of adjustments encountered during the transition from high school to college, including escalated responsibilities, heightened autonomy, relocation to unfamiliar locales, the pursuit of belonging and acknowledgment, self-actualization, and the formulation of future career trajectories. Throughout their academic journey spanning undergraduate and graduate studies, these stressors may detrimentally impact students’ well-being, precipitating manifestations of anxiety, depression, and stress (Demenech et al., 2021). Additionally, they may precipitate the onset of eating disorders (EDs) and alterations in dietary habits, with a particular prevalence among female students and those enrolled in health-related disciplines (Trindade et al., 2019). Thus, young adults are in a period that requires attention regarding their eating behavior.

On March 11, 2020, the World Health Organization (WHO) declared the COVID-19 epidemic, a disease caused by Sars-Cov-2 (WHO, 2020), and one of the measures suggested to curb the spread of the virus was social isolation. As a result, millions of Brazilians underwent a change in their routine, staying for a longer time at home. This pandemic situation contributed to increased levels of anxiety and stress, causing changes in behavior, including eating (Hill et al., 2022). Stress makes people overeat, especially seeking sugary foods and comfort foods (Yılmaz & Gökmen, 2020), as much as carbohydrates increase the production of serotonin, which may have a positive effect on mood (Muscogiuri et al., 2020). Therefore, this study seeks to achieve a better understanding of the eating behavior of university students, especially at this unusual moment, being the first to conduct this analysis in Brazil and the first to analyze intuitive eating in university students during the pandemic. The aim of this study is to evaluate the level of IE among university students in Brazil during the COVID-19 pandemic, with the specific objectives of verifying the association between intuitive eating and BMI, the situation of confinement, and the type of housing unit, the relationship between mental and eating disorders and intuitive eating, and the relationship between the male and female sexes with intuitive eating. The hypothesis was that intuitive eating during the pandemic would be lower compared to previous periods and that there would be an association between intuitive eating and social isolation.

## Method

### Design

This is a cross-sectional, quantitative study, conducted by means of an online questionnaire in Higher Education Institutions in Brazil. It was carried out with students from higher education institutions during the quarantine period.

### Data collection

Data collection was performed online by anonymously completing a form created with the Qualtrics survey software. It took place in the period from September 2020 to September 2021, by means of promotion on social media (Instagram, Facebook, Research Gate, and WhatsApp), and by sending emails to researchers and coordinators of university programs, university centers, and public and private universities in Brazil.

### Sample

The sample comprised undergraduate and graduate students from higher education institutions in Brazil with a minimum age of 18 years, of both sexes. The survey was answered by means of an online questionnaire. After excluding participants who failed to meet the inclusion criteria, including 7 pregnant women, due to their distinct eating behavior (Daundasekara et al., 2017) those who imposed constraints on free and informed participation; and those who responded to less than 54% of the questionnaire, as this would not include the IES-2 (1123), the final sample consisted of 1335 participants. Of these, 82.17% were females and 51% lived in the Southern Region of Brazil. The mean age was 26.12 ± 7.9 years, 41.4% were full-time students, 26.7% were studying nutrition, and 12.6% were in graduate programs. There was a higher prevalence of individuals with normal weight (57.58%), followed by overweight (24.14%), obese (12.38%), and underweight (5.69%) individuals. The mean BMI was 24.46 ± 4.9 kg/m^2^.

### Instruments

For data collection, the following instruments were used:

#### Questionnaire for characterizing the sample

The collected data encompassed the following variables: gender (female and male), pregnancy status, date of birth, marital status, email address, state and city of residence, household composition, level of education, institution of study, program enrolled in, original program modality (before the pandemic), original program schedule (before the pandemic), current occupation, self-reported weight (kg), self-reported height (cm), socioeconomic status, food allergies and/or intolerances, chronic illnesses, self-reported mental health status, and self-reported eating disorder presence. Furthermore, weight and height were obtained from the participant’s self-report (Freitas et al., 2020; Silveira et al., 2005). BMI was calculated by dividing weight by height squared, then classified according to the criteria recommended by the World Health Organization (WHO) for adults, underweight (less than 18.5), normal weight (between 18.5 and 24.9) overweight (between 25 and 29.9), and obesity (equal to or above 30) (WHO, 2000).

#### Questionnaire for evaluating the confinement situation and type of housing unit

This questionnaire was created by the researchers themselves and the collected data were confinement situation (confined at home, confined at home, except for occasional travel (e.g., shopping, supporting family members), regular mobility outside the home (e.g., essential professions), total mobility, normal life, because I do not think I should be confined) and type of housing unit to which participants were confined (apartment *without* balcony and/or terrace, apartment *with* balcony and/or terrace, house *without* an outdoor space (backyard), house *with* an outdoor space (backyard), other).

#### Intuitive Eating Scale 2 (IES-2)

It is a 23-item self-report scale (Tylka & Kroon Van Diest, 2013), designed to assess the tendency to follow internal hunger and satiety cues, considering 4 dimensions: Body-Food Choice Congruence (BFCC), Reliance on Hunger and Satiety Cues (RHSC), Unconditional Permission to Eat (UPE) and Eating for Physical rather than Emotional Reasons (EPER), each representing a subscale. It was developed by Tracy Tilka, a psychologist from Ohio, in 2006 (Tylka, 2006), and translated, adapted, and validated for the Brazilian population by da Silva et al. (2018), who found Cronbach's alpha values ranging from 0.79 to 0.89 in their study. Each item was answered using a Likert scale where 1 means strongly disagree and 5 strongly agree. High scores indicated greater IE, ranging from 1 to 5. The calculation of each of the subscales and the total IE score was done by averaging the scores; however, items 1, 2, 4, 5, 9, 10, and 11 had a reverse score, that was, for calculation purposes, 1 was worth 5, and so on. For the result of each of the subscales, the mean of the following items was calculated: 1, 3, 4, 9, 16, and 17 for UPE, 2, 5, 10, 11, 12, 13, 14, and 15 for the EPER subscale, 6, 7, 8, 21, 22, and 23 for RHSC, and finally 18,19, and 20 for BFCC. McDonald’s omega index (McDonald, 1999) for the scale in this project was 0.868, showing high reliability.

### Data analysis

Analyses were performed using version 21.0 of SPSS (Statistical Package for Social Science), JASP version 0.15, and G*Power version 3.1.9.7. The normality of numerical data distribution was verified by using the Kolmogorov–Smirnov test. Quantitative variables were described by mean and median and measure of dispersion (standard deviation and interquartile range).

One-way ANOVA with post hoc (Games-Howell) and Kruskal–Wallis tests were performed to analyze the difference between groups. In addition, a linear regression test was performed to verify the prediction for the confinement situation, BMI, and mental and eating disorders in IE all variables were included in the same prediction model. The analyses were performed considering a significance level of 95% (*p* < 0.05). To measure the effect size (Wassertheil & Cohen, 1970), an *F*-test was performed, followed by a power analysis performed using the GPower software version 3.1.9.7 (Faul et al., 2007) for Windows.

### Compliance with ethical standards

The study was approved by the Pontifical Catholic University of Rio Grande do Sul Research Ethics Committee, the CAAE number is 12444019.8.0000.5336, as available on *Plataforma Brasil*. Before starting the survey, all participants read and agreed to the free and informed consent form, which contained general information about the project, the aim, and the possible benefits and discomforts. After completing the questionnaire, participants received an infographic containing an explanation for each of the scales used. The contact details of the investigator in charge were provided for clarification if there was any doubt.

## Results

### Sample characterization

To assess the pandemic situation, they were asked about their confinement situation and type of housing unit. Most reported being confined at home with occasional trips, for example, for shopping or supporting family members (58.65%). Furthermore, 55.5% spent the pandemic period confined in a house with an outdoor area, for example, with a yard, and living with their family (69%). While the majority of students did not exhibit mental (67.2%) and eating disorders (91.9%), among those who reported experiencing any mental disorder (32.8%), a significant proportion (21.2%) reported experiencing anxiety. Moreover, compulsive eating disorder was self-reported by 4.7% of the participants, as indicated in Table 1.

**Table 1:** Sociodemographic characteristics of participants during the period from September 2020 to September 2021

Variable	Results
Sample	*N* = 1335
Gender	1.097 women (82.17%)
Age	Median 23.00 (21–29) years
	Mean 26.12 ± 7.9 years
Region of Brazil	
South	51%
Southeast	25.2%
North	7%
Northeast	13.8%
Center-West	3%
BMI	Median 23.53 (21.09–26.7)
	Mean 24.46 ± 4.9 kg/m^2^
Occupation	
Full-time student	41.42%
Student and worker	39.7%
Student and trainee	18.87%
Income	
None	1.64%
Up to 1 minimum wage (up to BRL 954.00)	6.14%
1 to 3 minimum wages (from BRL 954.00 to BRL 2,862.00)	29.43%
3 to 6 minimum wages (from BRL 2,862.00 to BRL 5,724.00)	28.68%
6 to 9 minimum wages (from BRL 5,724.00 to BRL 8,586.00)	13.33%
9 to 12 minimum wages (from BRL 8,586.00 to BRL 11,448.00)	8.46%
12 to 15 minimum wages (from BRL 11,448.00 to BRL 14,310.00)	4.49%
More than 15 minimum wages (more than BRL 14,310.00)	7.79%
BMI classification	
Underweight	5.8%
Normal weight	57.58%
Overweight	24.14%
Obesity	12.38%
Education level	
Technology program in progress	1.6%
Undergraduate program in progress	74.9%
Graduate program in progress	23.3%
Type of housing unit	
Apartment without a balcony and/or terrace	17.75%
Apartment with a balcony and/or terrace	20.22%
House without an outdoor area (yard)	5.76%
House with an outdoor area (yard)	55.5%
Other	0.74%
Lives with	
Alone	11.8%
Family	69%
Friends	3.5%
Student house	0.3%
Partner	15.2%
Confinement situation	
Confined at home	4.9%
Confined at home, except for occasional travels (e.g., shopping, support for family members)	58.6%
Regular mobility outside home (e.g., essential professions)	31%
Total mobility, normal life, because I don’t think I should be confined	5.3%
Mental disorder	
No	67.2%
Depression	6.5%
Anxiety	21.2%
Substance abuse/dependence on substance use, alcohol, and/or drugs	0.2%
Obsessive–compulsive disorder (OCD)	1%
Bipolar disorder	1.4%
Borderline disorder	0.4%
Post-traumatic stress disorder	0.4%
ADHD	1.4%
Other	0.5%
Eating disorder	
No	91.9%
Anorexia nervosa	0.4%
Bulimia nervosa	1.5%
Binge eating disorder	4.7%
Other non-specific eating disorders	1.5%

*SD* Standard deviation, *BMI* Body mass index, *ADHD* Attention deficit hyperactivity disorder, *OCD* Obsessive compulsive disorder

### Intuitive eating, university students, and pandemic

The mean of the scores on the scale was considered intermediate, around 3, with the lowest being observed on the Eating for Physical rather than Emotional Reasons scale. In the one-way ANOVA test between the type of housing unit and the confinement situation, a significant association was found between the type of housing unit (*p* = 0.01; effect size η^2^ = 0.01), as well as the confinement situation and the UPE (*p* = 0.04, effect size η^2^ = 0.07); however, the post-hoc test showed no difference between groups. The association between confinement situation/type of housing unit and the other subscales and the total score was not significant (Tables 2 and 3). The linear regression analysis between the confinement situation variable and IE, with the reference category total mobility, did not show a significant association.

**Table 2:** ANOVA tests between the IES-2 scale and the confinement situation

		Mean	Median	F (df;ss)	*p*
Total IES		3.2 ± 0.619	3.3 (2.8–3.7)	1.594 (3;1308)	0.189
	C	3.30 ± 0.58			
	CE	3.31 ± 0.62			
	RM	3.23 ± 0.61			
	TM	3.34 ± 0.66			
RHSC		3.3 ± 0.98	3.3 (2.6–4.0)	1.547 (3;1308)	0.201
	C	3.25 ± 1.04			
	CE	3.34 ± 0.97			
	RM	3.22 ± 0.97			
	TM	3.38 ± 1.01			
BFCC		3.6 ± 0.98	3.6 (3.0–4.3)	1.092 (3;1308)	0.351
	C	3.75 ± 0.97			
	E	3.65 ± 0.99			
	RM	3.57 ± 0.96			
	TM	3.73 ± 0.96			
EPER		3.12 ± 0.95	3.1 (2.5–3.7)	1.111 (3;1308)	0.344
	C	3.23 ± 0.99			
	CE	3.12 ± 0.97			
	RM	3.07 ± 0.91			
	TM	3.26 ± 1.00			
UPE		3.3 ± 0.58	3.3 (3.0–3.6)	2.723 (3;1308)	0.043
	C	3.24 ± 0.73			
	CE	3.37 ± 0.58			
	RM	3.29 ± 0.56			
	TM	3.23 ± 0.56			

*C* Confined at home, *CE* Confined at home, except for occasional travels, *RM* Regular mobility outside the home, *TM* Total mobility, *total IES-2* Total Intuitive Eating Scale score, *RHSC* Reliance on Hunger and Satiety Cues subscale, *BFCC* Body-Food Choice Congruence subscale, *EPER* Eating for Physical rather than Emotional Reasons subscale, *UPE* Unconditional Permission to Eat subscale, *df* degrees of freedom, *ss* sum of squares

**Table 3:** One-way ANOVA and Games-Howell post hoc tests IES-2 vs. type of housing

	Mean	F (df;ss)	*p*
Total IES		1.39 (4;2.13)	0.23
AS	3.25 ± 0.65		
AC	3.31 ± 0.61		
CS	3.16 ± 0.65		
CC	3.31 ± 0.60		
O	3.13 ± 0.69		
RHSC		1.86 (4;7.16)	0.11
AS	3.31 ± 1.0		
AC	3.34 ± 0.94		
CS	3.01 ± 1.02		
CC	3.32 ± 0.97		
O	3.4 ± 1.18		
BFCC		1.64 (4;6.3)	0.16
AS	3.64 ± 0.96		
AC	3.70 ± 0.95		
CS	3.39 ± 0.96		
CC	3.63 ± 0.99		
O	3.43 ± 0.87		
EPER		2.14 (4;7.8)	0.07
AS	2.99 ± 1.01		
AC	3.18 ± 0.97		
CS	2.98 ± 0.98		
CC	3.14 ± 0.92		
O	3.41 ± 0.79		
UPE		3.2 (4;4.37)	0.013
AS	3.33 ± 0.58^a^		
AC	3.24 ± 0.60^a,b^		
CS	3.42 ± 0.63^a^		
CC	3.36 ± 0.57^a,c^		
O	3.03 ± 0.42^a^		

*Total IES-2* Total Intuitive Eating Scale score, *RHSC* Reliance on Hunger and Satiety Cues subscale, *BFCC* Body-Food Choice Congruence subscale, *EPER* Eating for Physical rather than Emotional Reasons subscale, *UPE* Unconditional Permission to Eat subscale, *AS* apartment without a balcony and/or terrace, *AC* apartment with a balcony and/or terrace, *CS* house without an outdoor area (backyard), *CC* house with an outdoor area (backyard), *O* other, ^*a,b,c*^ different letters mean statistically different means in post hoc test, *df* degrees of freedom, *ss* sum of squares

### Intuitive eating and BMI

The IE scale showed a negative correlation with BMI, the results were presented in Table 4 (*p* < 0.001; effect size η^2^ = 0.14, large effect). The post-hoc test showed a significant difference between the underweight and normal-weight groups, and overweight and obese groups (Games-Howell *p* < 0.001), as well as between the normal-weight and overweight groups (Games-Howell *p* < 0.002), as shown in Table 4.

**Table 4:** One-way ANOVA and Games-Howell post hoc test IES-2 vs. BMI classification

	Mean	*F*	*p*
Total IES		68.44	< .001
0	3.67 ± 0.45^a^		
1	3.43 ± 0.55^b^		
2	3.09 ± 0.65^c^		
3	2.84 ± 0.57^d^		
RHSC		53.21	< .001
0	3.75 ± 0.81^a^		
1	3.52 ± 0.91^a^		
2	3.04 ± 0.95^b^		
3	2.64 ± 0.96^c^		
BFCC		16.81	< .001
0	3.73 ± 0.88^a^		
1	3.78 ± 0.93^a,b^		
2	3.47 ± 1.02^a,c^		
3	3.24 ± 1.02^b^		
EPER		43.66	< .001
0	3.60 ± 0.78^a^		
1	3.28 ± 0.89^a^		
2	2.88 ± 0.98^b^		
3	2.51 ± 0.92^c^		
UPE		13.64	< .001
0	2.66 ± 0.52^a^		
1	3.36 ± 0.58^b^		
2	3.22 ± 0.57^c^		
3	3.26 ± 0.52^b,c^		

*Total IES-2* Total Intuitive Eating Scale score, *RHSC* Reliance on Hunger and Satiety Cues subscale, *BFCC* Body-Food Choice Congruence subscale, *EPER* Eating for Physical rather than Emotional Reasons subscale, *UPE* Unconditional Permission to Eat subscale, *0* underweight, *1* normal weight, *2* overweight, *3* obesity, ^*a,b,c*^ different letters mean statistically different means

### Unconditional Permission to Eat (UPE) subscale

The UPE subscale showed a correlation with the BMI classifications, with the highest score being related to the lowest BMI (*p* < 0.001; effect size η^2^ = 0.031, moderate effect). In the post hoc comparison, there was a significant difference between the underweight and normal-weight groups and overweight and obese groups (*p* < 0.001), as well as between the normal-weight and overweight groups (*p* < 0.002), as shown in Table 4.

### Eating for Physical rather than Emotional Reasons (EPER) subscale

The EPER subscale showed an inverse correlation with BMI (*p* < 0.001; effect size η^2^ = 0.094, moderate effect), the only comparison that was not significant was between the underweight and normal-weight groups, as shown in Table 4.

### Body-Food Choice Congruence (BFCC) subscale

**Fig. 1:**
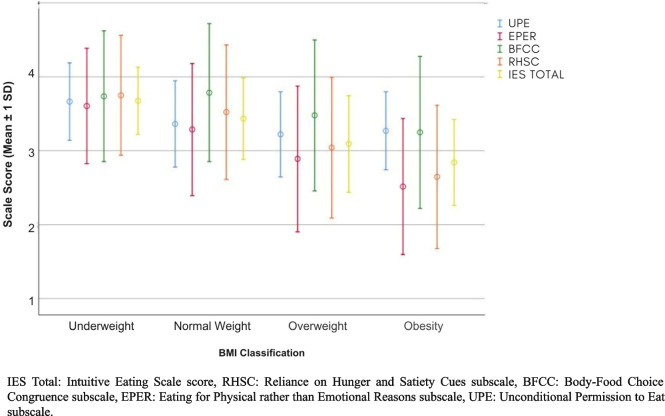
Association between BMI classification and intuitive eating in university students in Brazil

There was an inverse correlation between the BMI classifications and the BFCC subscale presented in Table 4 (*p* < 0.001; effect size η^2^ = 0.039, moderate effect), in which, according to the Games-Howell post-hoc test, there was a significant difference between the underweight and obese groups (*p* < 0.002), and between the normal weight, overweight and obese groups (*p* < 0.001), as shown in Table 4 and Fig. 1.

### Reliance on Hunger and Satiety Cues (RHSC) subscale

The RHSC subscale also showed an inverse correlation with BMI, as shown in Table 4 (*p* < 0.001; effect size η^2^ = 0.11 moderate effect), with a significant comparison between the low weight and overweight, obesity and between normal weight, overweight and obesity and between overweight and obesity, all with *p* < 0.001.

### Intuitive eating according to sex

In both sexes, the highest score was on the BFCC scale, the lowest for females was Eating for Physical rather than Emotional Reasons, and for males, it was Reliance on Hunger and Satiety Cues. In the ANOVA test, a significant difference was observed between sexes in the IE score and on the Eating for Physical rather than Emotional Reasons subscale, with males showing higher scores for both. Total IES-2: (*p* < 0.001; effect size η^2^ = 0.01 small effect), EPER: (*p* < 0.001; effect size η^2^ = 0.036 moderate effect). See Table 5.

**Table 5:** One-way ANOVA tests IES-2 vs. gender assigned at birth

		Mean	*F*	*p*
IES-2			13.33	< .001
	Male	3.4 ± 0.51		
	Female	3.2 ± 0.63		
RHSC			0.081	0.776
	Male	3.29 ± 0.90		
	Female	3.31 ± 0.99		
BFCC			0.182	0.67
	Male	3.6 ± 0.88		
	Female	3.63 ± 1.0		
EPER			48.31	< .001
	Male	3.5 ± 0.87		
	Female	3.0 ± 0.95		
UPE			0.001	0.97
	Male	3.3 ± 0.60		
	Female	3.3 ± 0.58		

*Total IES-2* Total Intuitive Eating Scale score, *RHSC* Reliance on Hunger and Satiety Cues subscale, *BFCC* body-food choice congruence subscale, *EPER* Eating for Physical rather than Emotional Reasons subscale, *UPE* Unconditional Permission to Eat subscale

### Intuitive eating and self-reported mental and eating disorders

In the linear regression analysis, individuals with bulimia and binge eating were observed to be significant predictors of IE. The population with eating disorders tends to score lower on the IE scale when there is no ED diagnosis. The individual who self-reported binge eating disorder had the lowest score. Regarding self-reported mental disorders, anxiety emerged as a significant predictor variable for IE compared to individuals without any diagnosed mental disorder. Anxiety scored lower on the IE scale compared to the reference category of individuals with no diagnosed mental disorder. Individuals with other mental and eating disorders were not significant predictors for IE. As shown in Table 6.

**Table 6:** One-way ANOVA and linear regression test IES-2 vs. eating and mental disorders

	One-way ANOVA			Linear regression		
	*F*	*p*	*R*^*2*^	*B*	Beta	*p*
	8.6	< .001	071			
ED				− 0.50	− .022	
Mental disorder				− 0.082	− 0.62	
Anorexia nervosa				.035	0.54	.18
Bulimia nervosa				− .58	− 0.079	< .001
BED				− .66	− 0.169	< .001
Other ED				− .21	− 0.013	.11
Depression				− .059	− 0.190	.4
Anxiety				− .102	− 0.329	.016
Substance abuse				.35	− 0.018	.39
OCD				− .35	− 0.107	.051
Bipolar				− .18	− 0.122	.2
Borderline				− .14	− 0.046	.58
ADHD				.042	− 0.067	.77
PTSD				.12	− 0.030	.68
Other				.064	− 0.247	.79

*Total IES-2* Total Intuitive Eating Scale score, *ED* eating disorder, *OCD* obsessive–compulsive disorder, *ADHD* attention deficit hyperactivity disorder, *PTSD* post-traumatic stress disorder, *BED* binge eating disorder

## Discussion

This study was the first to analyze the level of intuitive eating during the COVID-19 pandemic in Brazil. Unlike our study, no other study has assessed the confinement situation in relation to IE thus far. In this study, the mean found for IES-2 was 3.2 ± 0.6. It should be noted that, in an analysis of studies prior to the COVID-19 pandemic, the mean IE score found was slightly higher in female and male college students, respectively 3.38 ± 0.48 and 3.67 ± 0.52 (Tylka & Kroon Van Diest, 2013), and in the study by Barad et al. (2019), the median was 3.4 (3.3,3.7) in men and 3.4 (3.1,3.7) in women. Although an association was found between IE and the confinement situation, as well as the type of housing unit, a decrease in IE was observed during the data collection period of this research, which may be related to other factors, such as anxiety and stress.

Only two studies (Jackson et al., 2021; Sanlier et al., 2021) assessed IE during the pandemic. The study conducted in Turkey (Sanlier et al., 2021) with a sample of 1224 adults with a mean age of 27.5 ± 9.6 years had a mean IE score of 3.19 ± 0.34. In the United States (Jackson et al., 2021), a study conducted with 400 adults showed a total score on the IE scale of 3.1 in the group that reported feeling increasingly bored or that was eating out of boredom during the pandemic period, furthermore, our findings align with those reported in previous studies.

One of the hypotheses would be that the confinement situation was associated with IE levels during the COVID-19 pandemic in Brazil; however, in the ANOVA test, the only significant association was related to the unconditional permission to eat (UPE), albeit with a moderate effect size (η^2^ = 0.07). When the linear regression test was carried out using the total mobility variable as the reference category, no association was observed between the two variables. Therefore, the alternative hypothesis was rejected, since the confinement situation proved to be unrelated to the change in the IE of participants. In the ANOVA test for the variable type of housing, unit, a significant association was also seen only with the UPE subscale, but no significant association was found in the post hoc Games-Howell test.

The UPE subscale was associated with the confinement situation and the type of housing unit. To better understand this concept, the UPE does not refer to eating more foods that are not nutritious or eating greater amounts, but rather eating without classifying food in a dichotomous manner, as good or bad, maintaining a neutral relationship with food (Tylka, 2006). Nevertheless, studies have shown increased consumption of comfort foods at the time of confinement, as previously mentioned, which may be related to a greater UPE, in as much as food is associated with pleasure and directly connected to one’s mood (Özden & Parlar Kiliç, 2021; van Strien et al., 2019). At a time when negative emotions on the rise, such as depression, anxiety, and loneliness (Al-Musharaf, 2020; Brooks et al., 2020), it was observed that the greater permissiveness towards these foods was a way to seek comfort during confinement.

In this study, a high BMI was associated with lower scores for IE. A high BMI is a risk factor for a number of comorbidities, such as cardiovascular diseases (Dwivedi et al., 2020), and COVID-19 (Sattar et al., 2020; Yang et al., 2021). Over the years, overweight and obesity have been increasing in Brazil (Brazilian Institute of Geography and Statistics [IBGE], 2019) and around the world (Blüher, 2019). We found associations between a low BMI and high IE scores, as well as a high BMI and low IE scores, a result also found in other studies (Craven & Fekete, 2019; Özkan & Bilici, 2021). This indicates that eating intuitively could be a tool to maintain a stable body mass, some hypotheses that may explain this result are that people with more adipose tissue create resistance to leptin and insulin in view of the inflammatory process (Jéquier, 2006; Koleva et al., 2013; Yadav et al., 2013). Moreover, they have a mutation in the ob/ob gene or in the ob receptor that encodes leptin (Rohde et al., 2019), as well as a reduction in the dopamine receptor (D2R), presenting changes in their hunger and satiety cues (Volkow et al., 2013). However, further clinical studies must be conducted to assess these associations. Additionally, despite intuitive eating being found to be higher in individuals with low weight, it's important to note that a small sample of individuals with low weight (5.8%) may not adequately represent the diversity of the population in this condition and its relationship with intuitive eating.

College students who were obese had lower scores on the EPER subscale, suggesting the possibility of emotional eating, which is defined as overeating in the presence of negative emotions. Eating is known to involve physiological (homeostatic), social, and psychological issues (Alonso-Alonso et al., 2015). Consumption is often marked by the overlapping of non-homeostatic factors, which is known as emotional eating. Emotional eating lies within a healthy eating behavior and is dependent on the situation and the intensity of the emotion. People who are more prone to emotional eating are more likely to put on weight, in view of higher caloric intake and more palatable foods, such as sugar, salt, and fat, as well as “snacking” as maladaptive strategies to regulate emotions (Dakanalis et al., 2023). There is a short-term improvement in the negative emotion, as there is an increase in serotonin (Muscogiuri et al., 2020; Rezitis et al., 2022), but this feeling does not last for long and is often followed by a feeling of guilt. The pandemic was a trigger for emotional eaters, as there was a surge in negative feelings, such as anxiety and depression; therefore, studies have shown an increase in emotional eating during this period (Cecchetto et al., 2021; Husky et al., 2020), as well as an increase in weight gain (Santana et al. 2021), a fact that is in agreement with the findings of this study with Brazilian university students.

The eating behavior changed during the COVID-19 pandemic. The studies that evaluated it during this period showed an increase in the amount of food consumed (Huber et al., 2021), mainly comfort foods (Özden & Parlar Kiliç, 2021), worsening of eating disorders for those who already had a history (Meda et al., 2021), increased emotional eating (Cecchetto et al., 2021), and increased internalization of thin ideals (Baceviciene & Jankauskiene, 2021) in the student population. All these behaviors are negatively associated with IE; however, at the same time, there were studies that reported a decreased consumption of ultra-processed food in men and a decline in unhealthy eating habits in both sexes, as well as an increase in cooking (Baceviciene & Jankauskiene, 2021; Tribst et al., 2021).

The hypothesis that women would have lower IE than men was confirmed, a fact that supports other studies that highlight the greater vulnerability of women to eating and mental health disorders (depression, anxiety, and stress) (De Lima Braga et al., 2013; Demenech et al., 2021), as well as a higher obesity rate (IBGE, 2021). Moreover, women are subject to greater aesthetic and social pressure, which puts a cognitive restriction on eating (Rounsefell et al., 2020), a fact that prevents varied and nutritive food consumption. The double burden (IBGE, 2021), the wage gap (29.6% less than males) (IBGE, 2020), the pressure due to aesthetic standards (Baceviciene & Jankauskiene, 2021), sexual harassment and gender violence, which was more prevalent among women during the pandemic (IBGE, 2021), were aspects that made women more vulnerable to negative physical and mental health outcomes. This whole context may lead to emotional eating as a coping mechanism, resulting in higher rates of this behavior in females compared to males.

IE is known to be negatively related to eating disorders (Linardon et al., 2021). In this study was noted that both compulsive eating disorder and bulimia nervosa were correlated with lower scores on the IE scale. This observation suggests an association between higher intuitive eating (IE) scores and a reduced likelihood of developing eating disorders. Further longitudinal research is needed to explore the temporal relationship between IE scores and the development of eating disorders.

This is the first study assessing IE during the pandemic in university students from across Brazil. It is of paramount importance, since the population requires attention with regard to their eating behavior in view of their greater tendency to develop EDs (Trindade et al., 2019). In addition, the pandemic brought major changes to students, as classes adopted an online format (Husky et al., 2020). Thus, this study contributed to the understanding of the eating behavior of students, especially in this unusual moment.

The high rate of answers by females, as well as the non-inclusion of genders other than the binary ones in the form, may be limiting factors for the survey. However, we should consider that most studies conducted through online questionnaires during the pandemic obtained a sample that mostly comprised females (Baceviciene & Jankauskiene, 2021; Huber et al., 2021; Meda et al., 2021; Özden & Parlar Kiliç, 2021). Furthermore, women are the majority of university students in Brazil (IBGE, 2021). Another limitation was that the study was carried out cross-sectionally and was not able to determine cause and effect. Additionally, it should be noted that another limitation of the study was that the sample was composed largely of the southern region of Brazil and nutrition students.

The use of self-reported weight and height to calculate the BMI of participants may lead to changes in values; however, with the increase in studies conducted online, especially during the pandemic, self-reported weight and height are being increasingly used, and there are references showing that these are as reliable as when measured in person (Freitas et al., 2020; Silveira et al., 2005). Finally, the population of university students in Brazil cannot be generalized to young adults, and a greater understanding of IE in other cultures and countries is needed.

## Conclusion

The confinement situation and the type of housing unit exhibited significant associations with the UPE in university students in Brazil during the COVID-19 pandemic. Additionally, there was a negative association found between BMI, eating disorders, and IE. Moreover, IE levels were higher in males compared to females. The findings suggest an association between IE and better health outcomes, including lower rates of eating disorders, anxiety, depression symptoms, and BMI. Given the obesity epidemic, such an approach may be viable in prevention strategies and mapping risk factors. As IE brings about behavioral change, impacting lifestyle habits, and is cheaper, its applicability in public health practices would be interesting.

## Data Availability

The datasets generated and analyzed during the current study are not publicly available due to the no authorization of the participants but are available from the corresponding author on reasonable request.

## Abbreviations

IE: Intuitive Eating
BMI: Body Mass Index
EDs: Eating disorders
IES-2: Intuitive Eating Scale 2
BFCC: Body and Food Choice Congruence
RHSC: Reliance in Hunger and Satiety Cues
UPE: Unconditional Permission to Eat
EPER: Eating for Physical rather than Emotional Reasons
OCD: Obsessive–compulsive disorder
ADHD: Attention deficit hyperactivity disorder
PTSD: Post-traumatic stress disorder
BED: Binge eating disorder
